# miR-27b Targets KSRP to Coordinate TLR4-Mediated Epithelial Defense against *Cryptosporidium parvum* Infection

**DOI:** 10.1371/journal.ppat.1002702

**Published:** 2012-05-17

**Authors:** Rui Zhou, Ai-Yu Gong, Alex N. Eischeid, Xian-Ming Chen

**Affiliations:** Department of Medical Microbiology and Immunology, Creighton University Medical Center, Omaha, Nebraska, United States of America; University of Virginia Health System, United States of America

## Abstract

*Cryptosporidium* is a protozoan parasite that infects the gastrointestinal epithelium and causes a diarrheal disease. Toll-like receptor (TLR)- and NF-κB-mediated immune responses from epithelial cells, such as production of antimicrobial peptides and generation of reactive nitrogen species, are important components of the host's defense against cryptosporidial infection. Here we report data demonstrating a role for miR-27b in the regulation of TLR4/NF-κB-mediated epithelial anti-*Cryptosporidium parvum* responses. We found that *C. parvum* infection induced nitric oxide (NO) production in host epithelial cells in a TLR4/NF-κB-dependent manner, with the involvement of the stabilization of inducible NO synthase (iNOS) mRNA. *C. parvum* infection of epithelial cells activated NF-κB signaling to increase transcription of the miR-27b gene. Meanwhile, downregulation of KH-type splicing regulatory protein (KSRP) was detected in epithelial cells following *C. parvum* infection. Importantly, miR-27b targeted the 3′-untranslated region of KSRP, resulting in translational suppression. *C. parvum* infection decreased KSRP expression through upregulating miR-27b. Functional manipulation of KSRP or miR-27b caused reciprocal alterations in iNOS mRNA stability in infected cells. Forced expression of KSRP and inhibition of miR-27b resulted in an increased burden of *C. parvum* infection. Downregulation of KSRP through upregulating miR-27b was also detected in epithelial cells following LPS stimulation. These data suggest that miR-27b targets KSRP and modulates iNOS mRNA stability following *C. parvum* infection, a process that may be relevant to the regulation of epithelial anti-microbial defense in general.

## Author Summary

MicroRNAs (miRNAs) are small non-coding RNAs that regulate gene expression at the posttranscriptional level. Accumulating data indicate that miRNAs are an essential part of the complex regulatory networks that control various cellular processes, including host antimicrobial immune responses. Toll-like receptors (TLRs) play an essential role in the activation of innate immunity by recognizing specific patterns of microbial components and activating downstream intracellular signaling pathways, including NF-κB. However, the role of miRNAs in the regulation of TLR/NF-κB-mediated epithelial antimicrobial defense is still unclear. *Cryptosporidium* is a protozoan parasite that infects the gastrointestinal epithelium in humans. Here, we show that KSRP, an RNA-binding protein and a key mediator of mRNA decay, is a target for miR-27b. Infection by *Cryptosporidium parvum* activates TLR4/NF-κB signaling and increases miR-27b expression, causing a suppression of KSRP in infected host epithelial cells. Functionally, downregulation of KSRP stabilizes iNOS mRNA and promotes epithelial production of nitric oxide, a molecule with antimicrobial activity. Therefore, miR-27b confers TLR4/NF-κB-mediated epithelial cell anti-*Cryptosporidium parvum* defense though regulating KSRP. Our study provides a new area of exploration for fine-tuning TLR/NF-κB-mediated host reactions in response to microbial challenge.

## Introduction

*Cryptosporidium,* a zoonotic parasite of the phylum *Apicomplexa*, is found in 65% to 97% of surface water in the United States. This parasite is resistant to standard disinfection applied to drinking water and has been recognized as the leading cause of waterborne disease outbreaks worldwide [1]. *Cryptosporidium parvum* and *C. hominis* are the most common species in humans [1]. Infection by *C. parvum* causes an acute, self-limited diarrheal disease in immunocompetent individuals, but causes life-threatening syndromes in immunocompromised patients. Once ingested, *C. parvum* oocysts excyst in the gastrointestinal tract and release sporozoites to infect intestinal epithelial cells. *C. parvum* sporozoites can travel up to infect the epithelial cells lining the biliary tract, mainly in AIDS patients [1], [2]. Cryptosporidial infection is usually limited to epithelial cells at the mucosal surface, where the parasite undergoes both intracellular and extracellular life stages; thus, *C. parvum* is classified as a “minimally invasive” mucosal pathogen [1], [2].

Evidence from *in vitro* and *in vivo* studies indicates that both innate and adaptive immunity are involved in the resolution of cryptosporidiosis and resistance to infection [1]–[6]. The invasion of human gastrointestinal epithelial cells by *C. parvum in vitro* activates Toll-like receptor (TLR)/NF-κB signaling, resulting in the production and secretion of various cytokines and chemokines (e.g., IL-8 and IL-13), prostaglandin E2 (PGE_2_, which stimulates mucin production), and antimicrobial peptides (e.g., HBD-2, human beta-defensin 2) and nitric oxide (NO), which can kill *C. parvum* or inhibit parasite growth [4], [7]–[9]. Acquired resistance to cryptosporidial infection requires T-cells with the α/β type T-cell receptor [1], [2], [5]; in addition, the CD4+ T-cell subgroup has a protective role, in particular, for cryptosporidiosis among patients with AIDS [10]. Interestingly, adult mice are usually resistant to developing a disease when *C. parvum* oocysts are given by oral gavage, but a persistent *C. parvum* infection develops in IFN-γ-deficient mice [11]. Although lack of susceptibility to intestinal *C. parvum* infection was also demonstrated in C3H/HeJ TLR4-deficient mice, a delayed *C. parvum* clearance was recently identified in TLR4-deficient mice compared with wild-type animals using a mouse model of biliary cryptosporidiosis [12], [13]. Mice deficient in MyD88, an adapter protein critical for many TLR functions, showed an increase in parasite burden in the intestine after receiving *C. parvum* oocysts by oral gavage [6]. It has been postulated that TLR/NF-κB-mediated innate immune responses by epithelial cells are critical to the host's defense to cryptosporidial infection [1], [2], [4]–[6].

Both transcriptional and posttranscriptional mechanisms are involved in the regulation of epithelial antimicrobial defense. Until recently, most gene-expression studies have measured steady-state mRNA levels and, thereby, fail to account for different states of translational activation and different stabilities of individual mRNA species [14], [15]. The importance of posttranscriptional mechanisms is beyond simply determining the rate of mRNA translation and degradation. Mammalian cells have evolved posttranscriptional mechanisms to combinatorially regulate multiple mRNAs along a coordinated pathway, allowing cells to respond with unusual ability to environmental cues (referred to as a “posttranscriptional operon”) [14]. It is now apparent that the 3′-untranslated region (3′UTR) of mRNA can specifically control the rates of translation and degradation of individual mRNA [14], [16]. Several RNA-binding proteins, including the KH-type splicing regulatory protein (KSRP, also known as KHSRP), tristetraprolin (TTP) and Hu antigen R (HuR), recognize AU-rich elements (AREs) within the 3′UTRs of mRNAs and control their half-life time in the cytoplasm [15]–[17]. For example, many cytokine mRNAs have the AREs in their 3′UTRs and usually have a very short half-life (in the range of 10–30 min) in resting cells, effectively preventing cytokines' protein production. In activated cells, cytokine mRNA half-lives are in the range of several hours, accounting for a significant increase in protein production [14], [15]. In this regard, KSRP interacts with the mRNAs that have the AREs within their 3′UTRs, including mRNAs for inducible NO synthase (iNOS), IL-8 and cyclooxygenase-2 (COX-2), and is a key mediator of mRNA decay [18], [19]. Thus, the intracellular level of KSRP can potentially coordinate their stability in response to extracellular stimuli [18]. Indeed, induced production of IL-8, NO (synthesized by iNOS) and PGE_2_ (catalyzed by COX-2) has been implicated in host cells in response to a variety of pathogens, including *C. parvum* [4], [7], [9].

The 3′UTR is also critical to miRNA-mediated posttranscriptional gene regulation. In mammalian cells, miRNAs identify targets based on complementarities between each miRNA and the 3′UTRs of target mRNAs resulting in either mRNA cleavage or translational suppression of the genes [20]. In our previous studies, we demonstrated that activation of TLR4/NF-κB signaling in epithelial cells regulates transcription of miRNA genes to orchestrate host anti-*C. parvum* immune responses through modulation of miRNA-mediated posttranscriptional suppression [21]–[23]. Distinct alterations in the miRNA expression profile were detected in epithelial cells following *C. parvum* infection [22]. Activation of NF-κB signaling in infected cells regulates transcription of a subset of miRNA genes, including the *mir-125b1*, *mir-21*, *let-7i, mir-30b*, and *mir-23b-27b-24-1* cluster genes [22]–[24]. Functional manipulation of several NF-κB-dependent miRNAs in epithelial cells influences *C. parvum* infection burden *in vitro* [22], [23], raising the possibility that these miRNAs may directly regulate production of antimicrobial molecules important to epithelial defense. Nevertheless, the underlying mechanisms remain largely unknown.

In this study, we investigated the potential role of miR-27b in controlling epithelial cell anti-*C. parvum* defense. The data we report here demonstrate that epithelial cells increased production of NO following *C. parvum* infection in a TLR4/NF-κB-dependent manner. Following *C. parvum* infection, cells displayed increased stability of iNOS mRNA and a decreased level of KSRP through upregulation of miR-27b, a miRNA from the TLR4/NF-κB-dependent *mir-23b-27b-24-1* cluster gene in epithelial cells [22], [25]. Downregulation of KSRP and upregulation of miR-27b stabilized iNOS/IL-8/COX-2 mRNAs, contributing to epithelial anti-*C. parvum* defense. Therefore, miR-27b confers TLR4/NF-κB-mediated anti-*C. parvum* defense though regulating KSRP in epithelial cells. Because upregulation of miR-27b is dependent on TLR4/NF-κB signaling, it appears that miR-27b/KSRP-mediated iNOS/IL-8/COX-2 mRNA stability could be an important component of cell reactions in response to TLR4/NF-κB signaling in general.

## Results

### Production of NO in epithelial cells in response to *C. parvum* infection is TLR4- dependent and involves stabilization of iNOS mRNA

**Figure 1:**
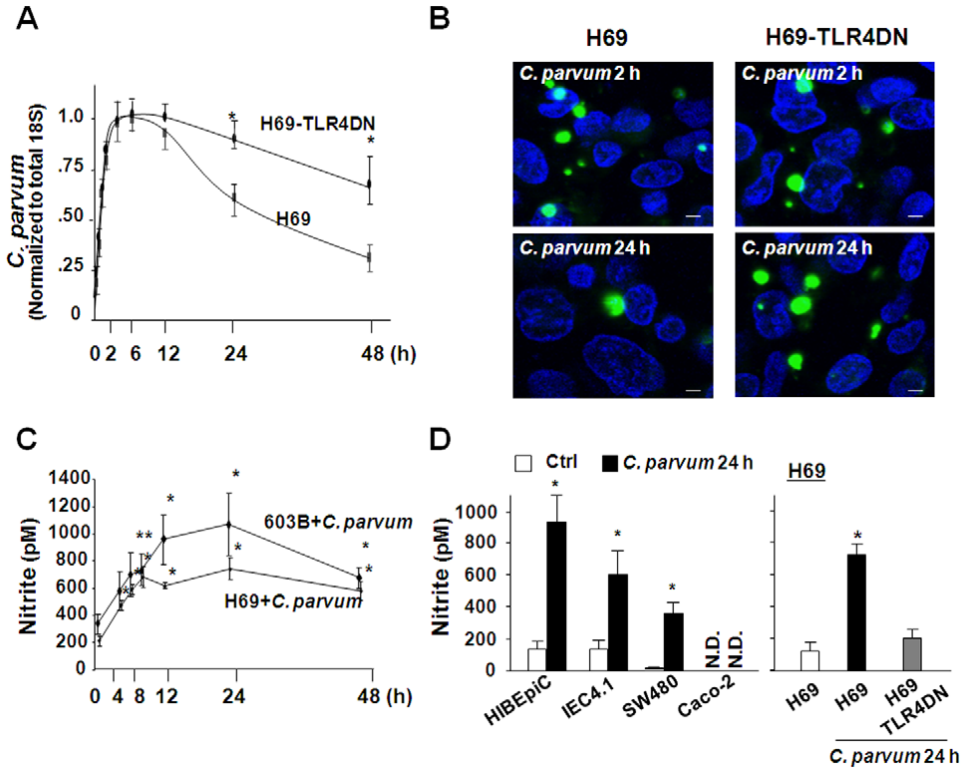
Severe impairment of epithelial anti-*C. parvum* defense in the absence of TLR4 *in vitro* and TLR4-dependent production of NO in response to *C. parvum* infection. A and B, Infection dynamics in cultured H69 cells and cells stably expressing TLR4DN after initial exposure to the same amount of *C. parvum* sporozoites, as assessed by real-time PCR for parasite 18S (A) or fluorescent microscopy (*C. parvum* stained by an antibody in green and cell nuclei by DAPI in blue in B). C and D, Effect of *C. parvum* infection on the production of NO in cells. H69 and 603B cells were exposed to *C. parvum* for up to 48 h, followed by nitrite measurement (C). IEC4.1, HIBEpiC, SW480, Caco-2 cells and H69 cells stably expressing TLR4DN were exposed to *C. parvum* for 24 h followed by nitrite measurement (D). *, *p*<0.05 vs non-infected cells (in A, C, and D). N.D. = not detectable. Bar = 1 µm.

We found that the *C. parvum* infection burden peaked at 2–4 h, then leveled off, and declined over time at 24 h and 48 h in H69 cells after initial exposure to *C. parvum* sporozoites, consistent with results from previous studies [22], [23]. In contrast, a higher infection burden was detected in cells stably expressing the TLR4-defective dominant negative (DN) mutant at 24 h and 48 h after the initial exposure to the same amount of sporozoites (Figure 1A). Notably, H69 cells and cells stably expressing TLR4DN showed a similar infection burden for the first 2–6 h after initial exposure to the parasite, suggesting that TLR4 signaling does not affect *C. parvum* attachment to and invasion of host cells, a process that completes within the first 2–4 h after exposure to host cells *in vitro* [1]. Immunofluorescent microscopy also revealed more parasites in H69 cells stably expressing TLR4DN mutant at 24 h after initial exposure to the same amount of sporozoites (Figure 1B). A significant increase in NO production was detected in H69 and mouse biliary epithelial (603B) cells following *C. parvum* infection for 4 h to 24 h. The production reached its peak at 24 h and slightly decreased but kept above the control level for up to 48 h after infection (Figure 1C). The increased production of NO was also detected by 24 h in several other TLR4-responsive epithelial cell lines following *C. parvum* infection (Figure 1D), including human intrahepatic biliary epithelial cells (HIBEpiC), human SW480 intestinal epithelial cells, and mouse intestinal epithelial cells (IEC4.1) [26]–[28]. Nevertheless, *C. parvum*-induced production of NO was not detected in Caco-2 cells (which do not express TLR4) [27]. No significant increase of NO was found in H69 cells stably expressing TLR4DN after *C. parvum* infection (Figure 1D).

**Figure 2:**
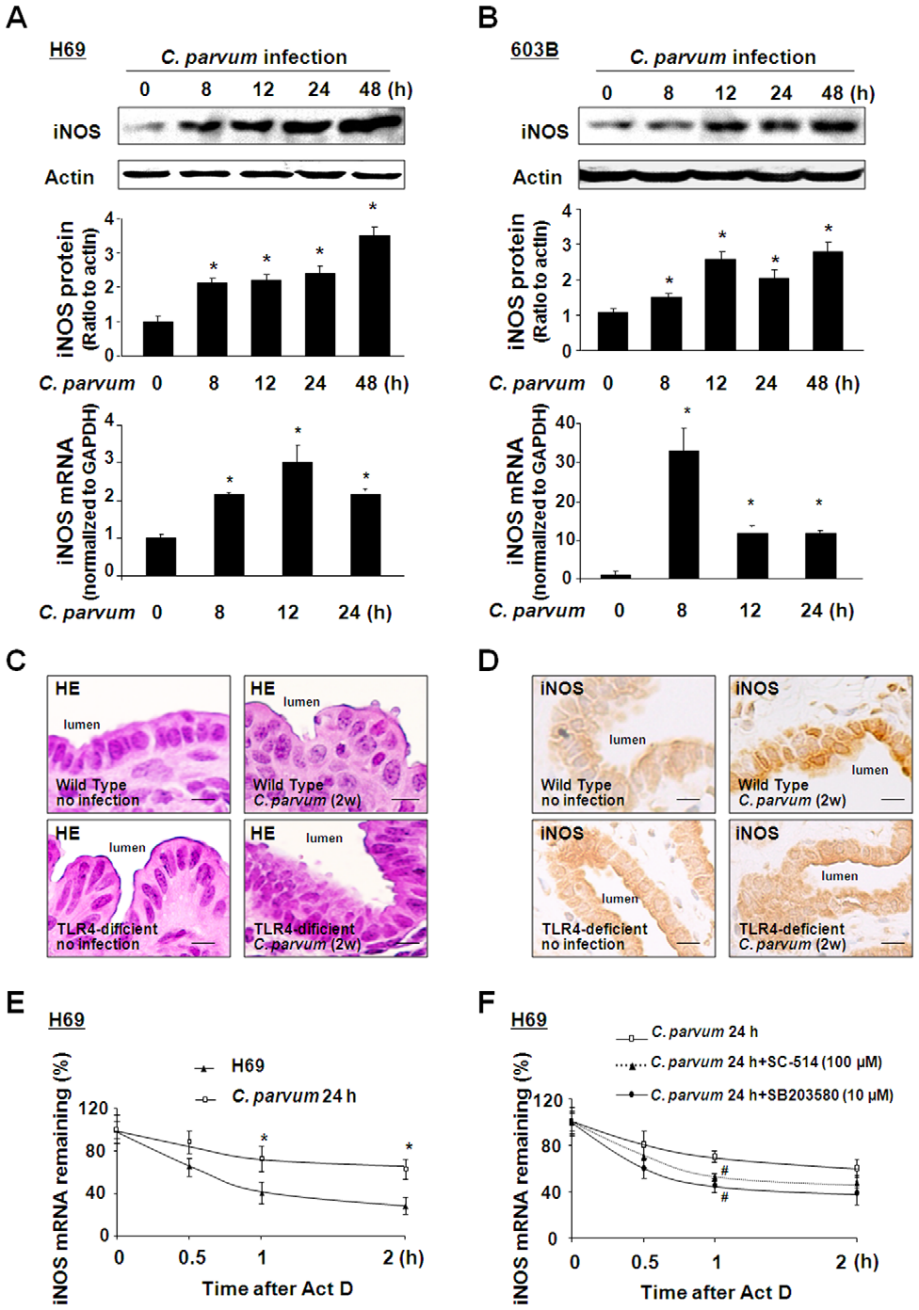
TLR4-dependent production of iNOS in response to *C. parvum* infection associated with stabilization of iNOS mRNA. A and B: H69 (A) and 603B (B) cells were exposed to *C. parvum* for up to 48 h, followed by Western blot for iNOS protein and real-time PCR analysis for iNOS mRNA. C: *C. parvum* oocysts were injected into the gallbladder of wild-type or TLR4-deficient mice. *C. parvum* infection in the intrahepatic bile ducts two weeks post-injection *was* detected by HE staining. Bar = 10 µm. D: Expression of iNOS was detected by immunohistochemistry in biliary epithelial cells from wild type mice or TLR4-deficient mice in the presence or absence of *C. parvum* injection for two weeks. Bar = 10 µm. E: Effects of *C. parvum* infection on the stability of iNOS mRNA in H69 cells. Cells were infected with *C. parvum* for 24 h. Actinomycin D (Act D) was then added and cells were collected for real-time PCR analysis. The stability of iNOS mRNAs was calculated, presented as the relative amount of mRNA to cells before Act D treatment. F: SC-514 and SB203580 partially blocked the stabilization of iNOS mRNA following *C. parvum* infection in H69 cells. Cells were infected with *C. parvum* for 24 h in the presence or absence of SC-514 (100 µM) or SB203580 (10 µM) following measurement of iNOS mRNA stability. Act D was then added and mRNA stability was measured as described above. Representative Western blot gels and quantification of iNOS mRNA levels from three independent experiments are shown. Densitometric levels of iNOS signals were quantified and expressed as the ratio to actin. *, *p*<0.05 vs non-infected cells (in A, B, and E); ^#^, p<0.05 *t*-test vs. infected cells without treatment with the inhibitors (in F).

NO production in epithelial cells is mainly regulated by iNOS [29]. Using Western blot, we found an increase of iNOS protein content in H69 and 603B cells following *C. parvum* infection (Figure 2A and 2B). A significant increase in iNOS mRNA was also detected in the cells following *C. parvum* infection (Figure 2A and 2B). Using a mouse model of biliary and intestinal cryptosporidiosis via gallbladder injection of *C. parvum* oocysts [30], we detected a significant increase in iNOS protein level in biliary epithelial cells from mice infected with *C. parvum* for two weeks, compared with the sham-infection control animals (Figure 2C and 2D). However, no significant increase of iNOS protein level was found in biliary epithelial cells from TLR4-deficient mice infected with *C. parvum* (Fig. 2D).

Expression of iNOS is controlled by transcriptional and posttranscriptional mechanisms involving gene transcription, RNA translation or turnover, and protein degradation [29]. To assess the effect of *C. parvum* infection on the stability of iNOS mRNA, H69 and 603B cells were infected by *C. parvum* for 24 h. The results of the half-life of iNOS mRNA, in the presence of actinomycin D, were measured by qRT-PCR. After actinomycin D treatment, the decay of iNOS mRNA in the *C. parvum*-infected H69 cells was relatively slow compared with the control uninfected cells (Figure 2E). The half-lives of iNOS mRNA in uninfected and infected cells were calculated to be T_1/2_ 45±8 min and T_1/2_ 150±20 min, respectively. Stabilization of iNOS mRNA was also detected in 603B cells following *C. parvum* infection (Figure S1). To test whether activation of TLR4 downstream pathways is involved in *C. parvum*-induced iNOS mRNA stabilization, we measured the mRNA stability in cells following *C. parvum* infection for 24 h in the presence of an IKK2 inhibitor (SC-514, which blocks NF-κB activation) [31] or a p38 MAPK inhibitor (SB203580). The stabilization of iNOS mRNA in cells following *C. parvum* infection was partially blocked by either SC-514 or SB203580 (Figure 2F). The half-lives of iNOS mRNA in infected cells in the absence and presence of SC-514, and SB203580 were calculated to be T_1/2_ 150±20 min, T_1/2_ 85±14 min, and T_1/2_ 65±12 min, respectively.

### *C. parvum* infection decreases KSRP expression in host epithelial cells through posttranscriptional mechanisms in a TLR4/NF-κB-dependent manner

**Figure 3:**
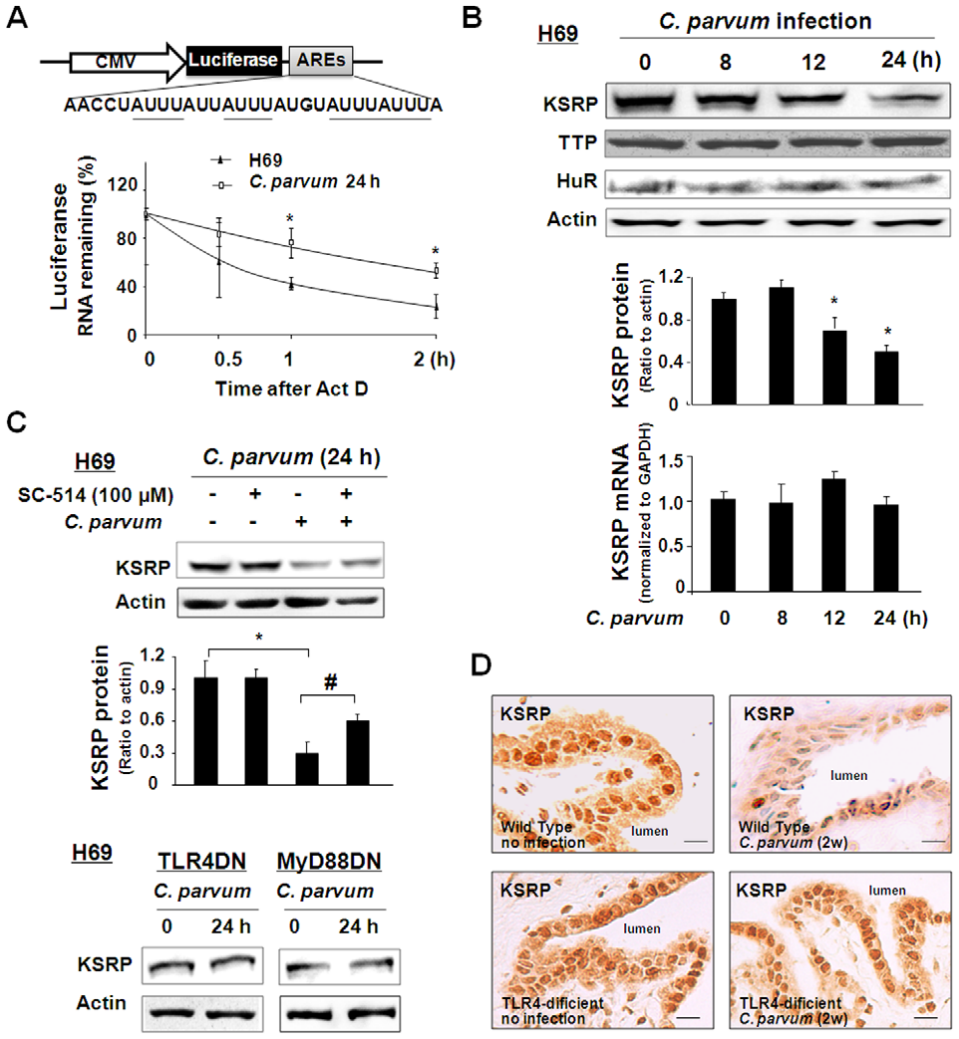
*C. parvum* infection decreases expression of KSRP protein content in epithelial cells by activation of the TLR4 signaling pathway. A: Effects of *C. parvum* infection on the stability of luciferase RNA in cells. ARE sequences were inserted downstream of a luciferase reporter on the pMIR-Report plasmid. H69 cells were transfected with the luciferase reporter carrying the AREs or the empty control vector for 24 h, and infected with *C. parvum* for an additional 24 h. Actinomycin D (Act D) was then added and cells were collected for real-time PCR analysis. The stability of luciferase RNAs was calculated, presented as the relative amount of RNA to cells before Act D treatment. B: H69 cells were exposed to *C. parvum* for up to 24 h, followed by Western blot for KSRP, TTP, and HuR proteins and real-time PCR analysis for KSRP mRNA. Representative Western blots from three independent experiments are shown. Densitometric levels of protein signals were quantified and expressed as the ratio to actin. C: H69 cells were exposed to *C. parvum* for 24 h in the presence or absence of SC-514 (100 µM), followed by Western blot for KSRP protein. SC-514 partially blocked the *C. parvum-*induced decrease of KSRP (upper). No decrease in KSRP protein was found in TLR4DN or MyD88DN cells following *C. parvum* infection, compared with the control non-treated cells (below). Representative Western blot gels and quantification of KSRP mRNA levels from three independent experiments are shown. Densitometric levels of KSRP signals were quantified and expressed as the ratio to actin. *, *p*<0.05 vs non-infected cells (in A–C); ^#^, p<0.05 *t*-test vs. infected cells (in C). D: Expression of KSRP was detected by immunohistochemistry in biliary epithelial cells from wild type mice or TLR4-deficient mice in the presence or absence of *C. parvum* injection for two weeks. Bar = 10 µm.

The 3′UTR of iNOS mRNA possesses the ARE sequence, and its stability can be regulated by RNA binding proteins such as KSRP, HuR, and TTP [19], [32], [33]. To test whether the ARE sequence is involved in stabilization of iNOS mRNA during *C. parvum* infection, we took a luciferase ARE reporter approach using a luciferase vector containing the ARE sequences, as previously reported by others [18]. H69 cells were transfected with the construct and then exposed to *C. parvum* for 24 h in the presence of actinomycin D. The levels of luciferase reporter RNA were measured by qRT-PCR. Degradation of the luciferase reporter RNA carrying the AREs was slowed down in the *C. parvum*-infected cells compared to that in the control cells (Figure 3A). The half-lives of luciferase RNA in uninfected and infected cells were T_1/2_ 62±12 min and T_1/2_ 130±15 min, respectively, suggesting the involvement of ARE-mediated RNA stability in cells following infection. We then measured the protein levels of KSRP, HuR, and TTP in H69 cells after exposure to *C. parvum* for up to 24 h. Using Western blot, a significant decrease in KSRP protein levels was detected in cells infected by *C. parvum* for 12 h and 24 h (Figure 3B). No significant change in TTP and HuR expression was detected in cells after exposure to *C. parvum* (Figure 3B). The decrease in KSRP protein level was also found in 603B, IEC4.1, SW480 and HIBEpiC cells following *C. parvum* infection for 24 h (Figure S2A–S2C). Interestingly, no significant change in KSRP mRNA levels was detected by real-time PCR analysis (Figure 3B and Fig S2A–S2C).

To test whether TLR4 signals are involved in *C. parvum*-induced KSRP expression, we tested the expression of KSRP in H69 cells stably transfected with the TLR4DN or MyD88DN [4], [23]. No decrease in KSRP protein was found in cells stably expressing TLR4DN or MyD88DN following *C. parvum* infection (Figure 3C). SC-514 also partially blocked the *C. parvum-*induced decrease of KSRP (Figure 3C). As shown in Fig. S2D, *C. parvum* infection not only decreased cytoplasmic KSRP levels, but also changed the nucleus KSRP levels in H69 cells. In addition, the decrease of KSRP expression in biliary epithelial cells was detected by immunohistochemistry in the livers of wild-type mice two weeks following *C. parvum* infection (Figure 3D). In contrast, no significant decrease in KSRP protein was found in the liver tissues from TLR4-deficient mice following *C. parvum* infection (Figure 3D).

### *C. parvum* infection activates TLR4/NF-κB signaling to increase miR-27b expression resulting in translational suppression of KSRP

**Figure 4:**
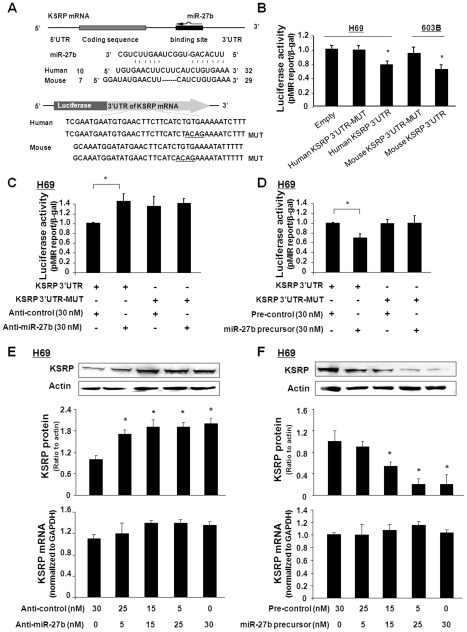
miR-27b targets KSRP 3′UTR, causing translational suppression. A: The schematic of KSRP mRNA shows one potential binding site for miR-27b in its 3′UTR. KSRP 3′UTR sequence covering the potential binding site was inserted into the pMIR-REPORT luciferase plasmid. Control plasmids with the mutant 3′UTR sequence were also generated for control. B, C, and D: Targeting of KSRP 3′UTR results in translational suppression. Cells were transfected with the pMIR-REPORT luciferase construct containing the miR-27b binding site in KSRP 3′UTR and treated with the anti-miR-27b or miR-27b precursor for 24 h, followed by luciferase analysis. E and F: Manipulation of miR-27b function results in reciprocal alterations in KSRP protein expression in H69 cells. Cells were treated with various doses of miR-27b precursor or anti-miR-27b for 48 h, followed by Western blot for KSRP protein and real-time PCR for KSRP mRNA. Representative Western blot gels and quantification of KSRP mRNA levels from three independent experiments are shown. Densitometric levels of KSRP signals were quantified and expressed as the ratio to actin.

The inconsistency in KSRP mRNA level with its protein content in cells following *C. parvum* infection suggests a potential posttranscriptional regulation of KSRP expression. To test whether miRNA-mediated posttranscriptional gene regulation is involved in this process, we used three algorithms (Targetscan 4.2 at http://www.Targetscan.org; MiRanda at http://www.microrna.org; and PicTar at http://pictar.mdc-berlin.de/) [34], [35] to screen potential KSRP-targeting miRNAs, by focusing on those miRNAs whose expression levels are altered in H69 cells following *C. parvum* infection, based on our previous microarray analysis [22]. We found that miR-27b has complementarity with KSRP 3′UTR, conserved for humans and mice (Figure 4A). No significant complementarity with the 3′UTR of HBD-2 mRNA for miR-27b has been identified. To elucidate whether miR-27b can bind to KSRP 3′UTR, resulting in translational suppression, we generated pMIR-REPORT luciferase constructs containing the KSRP 3′UTR with the putative miR-27b binding site (Figure 4A). pMIR-REPORT luciferase constructs containing the KSRP 3′UTR with a mutation at the putative miR-27b binding site (GTGA to ACAG) were generated as the controls (Figure 4A). We transfected cells with these reporter constructs, followed by assessment of luciferase activity 24 h after transfection. The transfection efficiency for the 3′UTR luciferase plasmids in H69 and 603B cells was from 20% to 40%, and, therefore, luciferase activity was normalized to the expression of the control β-gal construct. As shown in Figure 4B, a significant decrease in luciferase activity was detected in cells transfected with the KSRP 3′UTR construct containing the potential binding site, compared with the empty control vector. No change in luciferase activity was observed in cells transfected with the mutant KSRP 3′UTR construct, suggesting endogenous translational repression of the construct with the KSRP 3′UTR. In addition, anti-miR-27b markedly increased KSRP 3′UTR-associated luciferase reporter translation and did not impact the mutant KSRP 3′UTR construct in H69 cells (Figure 4C) and 603B cells (Figure S3A). In contrast, miR-27b precursors significantly decreased the luciferase reporter translation, and no change in luciferase activity was observed in cells transfected with the mutant KSRP 3′UTR construct in H69 cells (Figure 4D) and 603B cells (Figure S3B).

To test whether miRNA-mediated translational repression of KSRP is relevant to KSRP protein expression, we treated H69 and 603B cells with anti-miR-27b or miR-27b precursor for 48 h and then measured KSRP protein expression by Western blot. Transfection of H69 and 603B cells with anti-miR-27b caused a dose-dependent increase in KSRP protein content (Figure 4E and Figure S3C). No significant change in KSRP mRNA levels was found between the control cells and cells treated with anti-miR-27b (Figure 4E and Figure S3C). In contrast, a dose-dependent decrease in KSRP protein content was identified in cells treated with the miR-27b precursor (Figure 4F and Figure S3D). No significant change in KSRP mRNA levels was found between the control cells and cells treated with miR-27b precursor (Figure 4F and Figure S3D). No change in HBD-2 protein and mRNA levels was detected in cells following treatment with the miR-27b precursor or anti-miR-27b (data not shown).

**Figure 5:**
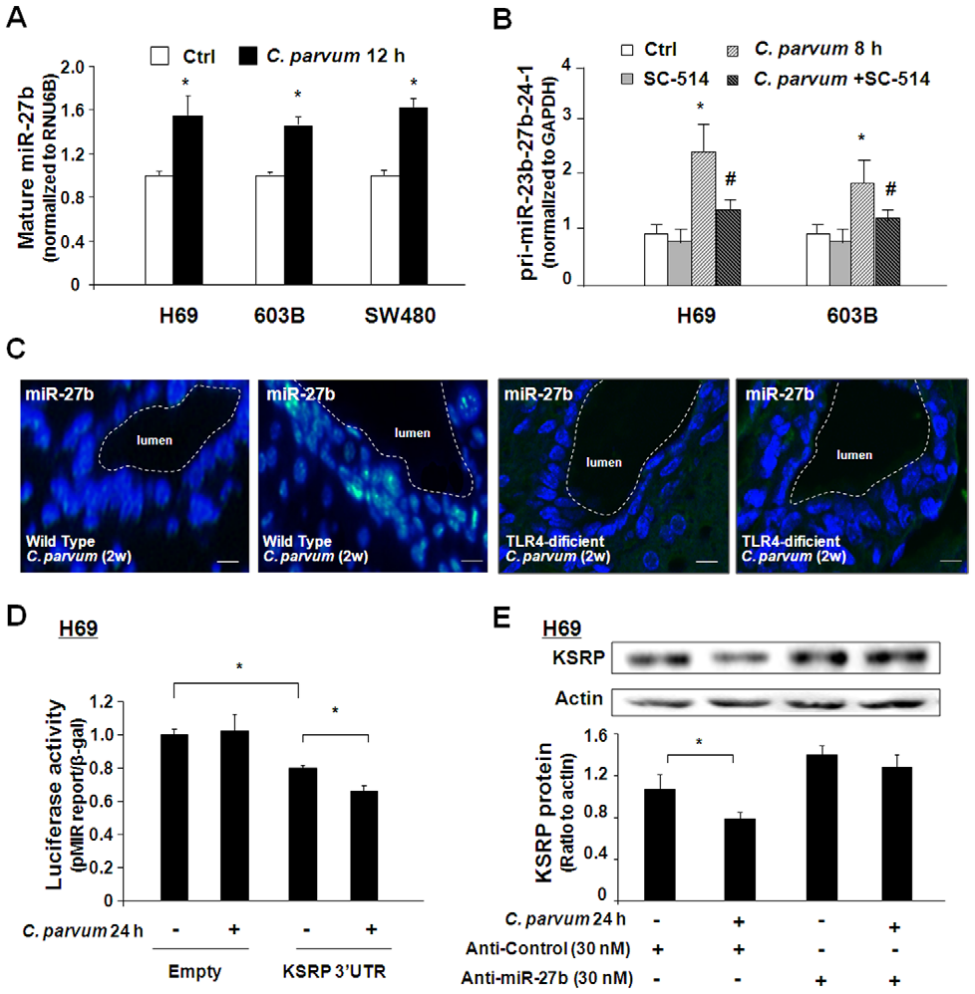
*C. parvum* infection increases miR-27b expression in epithelial cells in a TLR4/NF-κB-dependent manner. A: Alterations in mature miR-27b expression in H69, 603B and SW480 cells following *C. parvum* infection. Cells were exposed to *C. parvum* for 12 h and the level of mature miR-27b was obtained by real-time PCR, normalizing to snRNA RNU6B and relative to the control non-infected cells. B: Effects of NF-κB inhibition on *C. parvum*-induced upregulation of pri-miR-23b-27b-24-1 in H69 and 603B cells. The expression level of pri-miR-23b-27b-24-1 was quantified by real-time PCR following *C. parvum* infection for 8 h in the presence or absence of SC-514. C: Expression of miR-27b in biliary epithelial cells from wild type mice or TLR4-deficient mice in the presence or absence of *C. parvum* injection for two weeks. miR-27b was labeled in green by *in situ* hybridization using a DIG-conjugated probe and cell nuclei by DAPI in blue. D: *C. parvum* infection decreased KSRP 3′UTR-associated luciferase activity. H69 cells were transfected with the pMIR-REPORT luciferase construct containing the KSRP 3′UTR with the miR-27b binding sites and infected with *C. parvum* for 24 h, followed by luciferase analysis. E: Anti-miR-27b inhibited downregulation of KSRP protein induced by *C. parvum*. H69 cells were transfected with anti-miR-27b or anti-miR-control for 48 h and then exposed to *C. parvum* for 24 h followed by Western blot for KSRP. Representative Western blot gels are shown and densitometric levels of KSRP signals were quantified and expressed as their ratio to actin. *, p<0.05 *t*-test vs. non-infected cells (in A, B, D, and E); ^#^, p<0.05 *t*-test vs. infected cells (in B).

In our previous studies, we demonstrated that *C. parvum* infection increases miR-27b expression in H69 cells [22]. Using real-time PCR, we detected consistently a significant increase of miR-27b in H69, 603B, and SW480 cells following *C. parvum* infection (Figure 5A). miR-27b has been reported as a cluster miRNA sharing the same promoter with miR-23b and miR-24 [36]. We previously described pri-miR-23b-27b-24-1 as an NF-κB-dependent miRNA cluster in H69 cells [22], [25]. In agreement with previous results, the expression of pri-miR-23b-27b-24-1 increased in H69 and 603B cells following *C. parvum* infection, and SC-514 blocked *C. parvum*-induced increase in pri-miR-23b-27b-24-1 (Figure 5B). The increase of miR-27b expression level in biliary epithelial cells was detected in the livers of wild-type mice at two weeks following *C. parvum* infection by *in situ* hybridization (Figure 5C). In contrast, no significant increase in miR-27b was found in the liver tissues from TLR4-deficient mice following *C. parvum* infection (Figure 5C).

Since miR-27b can target KSRP 3′UTR and induces translational suppression of KSRP, *C. parvum* infection should induce an aggravation of miRNA-mediated KSRP translational suppression through upregulation of miR-27b. To test this possibility, we transfected cells with the pMIR-REPORT luciferase construct containing the KSRP 3′UTR with the miR-27b binding site, followed by exposure to *C. parvum* for 24 h. *C. parvum* infection decreased the KSRP 3′UTR-associated luciferase activity (Figure 5D). Anti-miR-27b inhibited the downregulation of KSRP protein in H69 and SW480 cells induced by *C. parvum* infection (Figure 5E and Figure S4). Coupled with the upregulation of miR-27b in infected cells, the above data suggest that aggravation of miR-27b-mediated translational repression is involved in *C. parvum*-induced KSRP protein expression.

### miR-27b regulates the stabilization of iNOS mRNA through targeting KSRP

**Figure 6:**
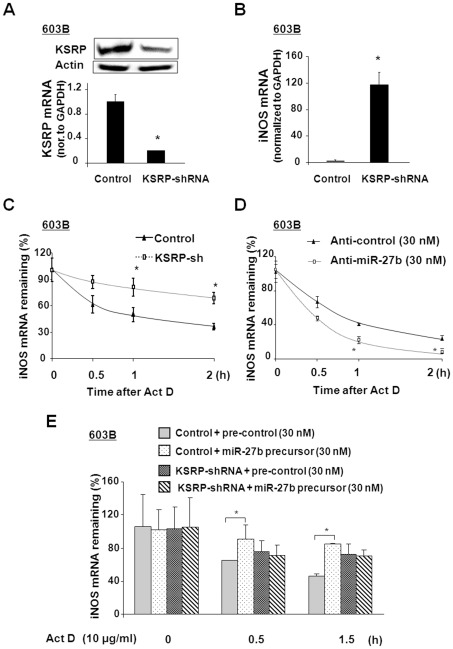
KSRP and miR-27b are involved in the posttranscriptional regulation of iNOS expression. A and B: Effects of KSRP knockdown on KSRP mRNA/protein or iNOS mRNA expression in 603B cells. Cells stably expressing KSRP shRNA were selected by Western blot for KSRP protein and real-time PCR analysis for KSRP mRNA. Downregulation of KSRP mRNA/protein level (A) and upregulation of iNOS mRNA level (B) were detected in cells stably expressing KSRP shRNA, compared with the control 603B cells. C: Effects of KSRP knockdown on iNOS mRNA stability in 603B cells. The stability of iNOS mRNAs was calculated in cells stably expressing KSRP shRNA and 603B cells following LPS stimulation for 2 h. D: effects of anti-miR-27b in iNOS mRNA stability in 603B cells. 603B cells were transfected with anti-miR-27b or control for 48 h. The stability of iNOS mRNAs was calculated in cells following LPS stimulation for 2 h. E: The impact of miR-27b precursor on iNOS mRNA stability was abolished in 603B cells stably expressing the KSRP shRNA. Cells stably expressing KSRP shRNA were transfected with miR-27b precursor or control for 48 h. The stability of iNOS mRNAs was measured after LPS stimulation for 2 h. *, p<0.05 *t-*test vs. the controls.

Previous studies suggested that KSRP regulates iNOS expression by destabilizing iNOS mRNA [19]. To determine whether KSRP plays a role in the regulation of iNOS expression, iNOS mRNA stability was detected in 603B cells stably expressing KSRP shRNA. A significant decrease in KSRP at the message and protein levels was determined in cells stably expressing KSRP shRNA, compared with the control 603B cells (Figure 6A). Accordingly, the levels of iNOS mRNA in these cells increased significantly (Figure 6B). As shown in Figure 6C, 603B cells stably expressing KSRP shRNA displayed a significant increase in iNOS mRNA stability. The half-lives of iNOS mRNA in control 603B cells and 603B stably expressing KSRP shRNA were calculated to be T_1/2_ 55±18 min and T_1/2_152±22 min, respectively.

Since KSRP is a target for miR-27b in epithelial cells, we predicted that miR-27b regulates iNOS mRNA stability by targeting KSRP. To test this possibility, iNOS mRNA stability was measured in 603B and H69 cells transfected with anti-miR-27b or miR-27b precursor. Anti-miR-27b showed a significant decrease in iNOS mRNA stability in 603B cells (Figure 6D) and H69 cells (Figure S5). The half-life of iNOS mRNA in 603B cells transfected with anti-control was T_1/2_ 50±12 min, and the half-life of iNOS mRNA in 603B cells transfected with anti-miR-27b was T_1/2_ 30±7 min. As shown in Figure 6E and Figure S5, miR-27b precursor increased iNOS mRNA stability. The half-lives of iNOS mRNA in 603B cells transfected with pre-control and miR-27b precursor were calculated to be T_1/2_ 60±14 min and T_1/2_ 160±28 min. Moreover, the impact of miR-27b precursor on iNOS mRNA stability was abolished in 603B cells stably transfected with the KSRP shRNA (Figure 6E).

### miR-27b and KSRP are involved in the regulation of epithelial defense against *C. parvum* infection

**Figure 7:**
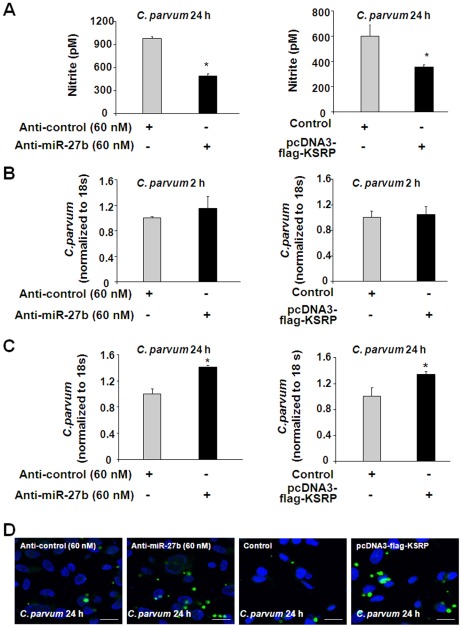
miR-27b and KSRP are involved in eradication of *C. parvum* infection through regulation of NO production. A: Effects of anti-miR-27b and KSRP overexpression on NO production in H69 cells. Cells were transfected with anti-miR-27b or pcDNA3-Flag-KSRP for 24 h, followed by exposure to *C. parvum* for an additional 24 h. Cell lyses were collected for nitrite measurement. B and C: H69 cells were transfected with anti-miR-27b, as well as pcDNA3-Flag-KSRP for 24 h. Cells were exposed to an equal number of *C. parvum* for 2 h, followed by extensive washing with culture medium. For determination of initial attachment and cellular invasion of *C. parvum*, cells were harvested immediately after washing and *C. parvum* was quantified by real-time PCR. To determine parasite burden after initial cell attachment and invasion, infected H69 cells were cultured for another 22 h after washing, followed by real-time PCR analysis. A similar number of parasites were detected in cells transfected with anti-miR-27b or pcDNA3-Flag-KSRP, after initial exposure to *C. parvum* for 2 h (B). Transfection of cells with anti-miR-27b or pcDNA3-Flag-KSRP increased *C. parvum* infection burden *in vitro* 24 h after initial exposure to the parasite (C). *, *p*<0.05 vs cells transfected with a non-specific control anti-miR or empty vector (in A and C). D: Effects of anti-miR-27b or pcDNA3-Flag-KSRP on *C. parvum* burden in H69 cells *in vitro* 24 h after initial exposure to the parasite, as assessed by immunofluorescent microscopy. *C. parvum* parasites were stained in green and nuclei in blue. Bar = 10 µm.

Since KSRP and miR-27b can regulate iNOS expression though regulating iNOS mRNA stability, KSRP and miR-27b should influence epithelial cell NO production and contribute to epithelial anti-*C. parvum* defense. We found a significant decrease in NO production in H69 cells transfected with anti-miR-27b or pcDNA3-flag-KSRP following *C. parvum* infection for 24 h (Figure 7A). To directly test whether miR-27b and KSRP are involved in epithelial defense against *C. parvum* infection, we assessed the parasite burden over time in H69 cells transfected with anti-miR-27b or pcDNA3-flag-KSRP. Cells were transfected with specific anti-miR-27b (60 nM) or pcDNA3-flag-KSRP and then exposed to a constant number of *C. parvum* sporozoites for 2 h, a model testing parasite attachment and cellular invasion [22], [23]. No change in the parasite burden was detected in any of the cultures (Figure 7B), suggesting that anti-miR-27b and KSRP overexpression do not affect initial parasite host cell attachment and cellular invasion. Next, we exposed cells transfected with anti-miR-27b (60 nM) or pcDNA3-flag-KSRP to *C. parvum* sporozoites for 24 h, a model testing the parasite proliferation that reflects host antimicrobial defense [22], [23]. We detected a significantly higher parasite burden in cells treated with the anti-miR-27b and cells transfected with the pcDNA3-flag-KSRP (Figure 7C). Increase of parasite burden 24 h after initial infection in H69 cells treated with anti-miR-27b or pcDNA3-flag-KSRP was further confirmed by immunofluorescent microscopy (Figure 7D), suggesting that miR-27b and KSRP are involved in the regulation of epithelial defense against *C. parvum* infection.

### miR-27b may play a role in the regulation of mRNA stability of inflammatory genes through targeting KSRP in epithelial cells in response to TLR4/NF-κB signaling in general

**Figure 8:**
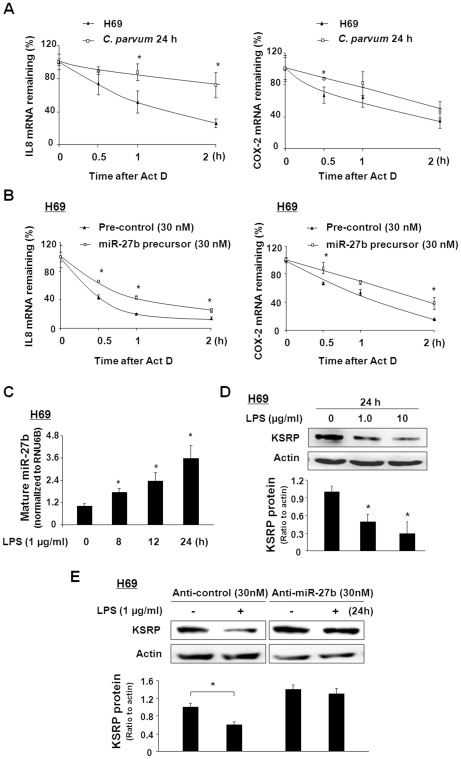
miR-27b may be involved in the regulation of mRNA stability of inflammatory genes through targeting KSRP in response to TLR4/NF-κB signaling in general. A: Effect of *C. parvum* infection on the stability of IL8 and COX-2 mRNA in H69 cells. Cells were infected with *C. parvum* for 24 h. Actinomycin D (Act D) was then added and cells were collected for real-time PCR analysis. The stability of mRNAs was calculated, presented as the relative amount of mRNA to cells before Act D treatment. B: Effects of miR-27b precursor in IL8 and COX-2 mRNA stability in H69 cells. H69 cells were transfected with miR-27b precursor or control for 48 h. The stability of mRNAs was calculated in cells following LPS stimulation for 2 h. C: Alterations in mature miR-27b expression in H69 cells after exposure to LPS for various periods of time, as assessed by real-time PCR. The level of mature miR-27b was obtained by normalizing the reactions to the level of snRNA RNU6B. Data are expressed as the amount of mature miR-27b in LPS-stimulated samples relative to the control non-stimulated samples. Results are representative of three independent experiments. D: H69 cells were exposed to LPS for 24 h followed by Western blot for KSRP. E: Anti-miR-27b inhibited the downregulation of KSRP protein in H69 cells induced by LPS stimulation. Cells were transfected with anti-miR-27b or anti-miR-control for 48 h and then exposed to LPS for 24 h, followed by Western blot for KSRP protein. Representative Western blot gels from three independent experiments are shown, and densitometric levels of KSRP signals were quantified and expressed as the ratio to actin. *, p<0.05 *t-*test vs. non-infected cells (in A and C). *, p<0.05 *t-*test vs. the controls (in B).

Given the fact that IL-8 and COX-2 mRNAs contain AREs in the 3′UTR [18], [37], we assessed IL-8 and COX-2 mRNA stability following *C. parvum* infection for 24 h. We detected an increase in the mRNA stability for IL-8 and COX-2 in H69 cells following *C. parvum* infection for 24 h (Figure 8A). The half-lives of IL-8 mRNA in uninfected and infected cells were calculated to be T_1/2_ 56±12 min and T_1/2_ 158±24 min, respectively. The half-lives of COX-2 mRNA in uninfected and infected cells were calculated to be T_1/2_ 70±18 min and T_1/2_ 112±24 min, respectively. Similar results were also observed in 603B cells following *C. parvum* infection (data not shown). Moreover, overexpression of miR-27b in H69 cells by miR-27b precursor increased the mRNA stability of IL-8 and COX-2 (Figure 8B). The half-lives of IL-8 mRNA in H69 cells transfected with pre-control or pre-miR-27b were T_1/2_ 42±8 min and T_1/2_ 70±10 min, respectively. The half-lives of COX-2 mRNA in H69 cells transfected with pre-control or pre-miR-27b were calculated to be T_1/2_ 65±17 min and T_1/2_ 108±18 min, respectively.

To test whether TLR4/NF-κB-mediated miR-27b regulates mRNA stability by targeting KSRP in general, we measured the expression of miR-27b and KSRP expression in H69 cells in response to LPS. Consistent with data from a previous report [25], we detected a significant increase in its mature form in cells following LPS stimulation (Figure 8C). Accordingly, LPS treatment caused a marked decrease in the content of KSRP protein in H69 cells in a dose-dependent manner (Figure 8D). No changes in KSRP mRNA levels were detected in LPS-treated cells, compared with that in the untreated control cells (data not shown). The anti-miR-27b inhibited the downregulation of KSRP protein in H69 cells induced following LPS stimulation (Figure 8E). In contrast, the anti-miR-control showed no effects. Thus, transfection of anti-miR-27b attenuated the downregulation of KSRP protein induced by LPS.

## Discussion

Production of NO by iNOS during infection plays an important role in epithelial innate immunity [38]. NO and NO-releasing compounds have been demonstrated to mediate host protection against a growing list of protozoan and helminth parasites *in vitro* and in animal models, either through direct parasite killing or by limiting parasite growth [9], [39]. The best known parasitic macromolecular targets for NO (-donors) are cysteine proteases, which are relevant in several aspects of the parasite life cycle and parasite-host relationships, and appear to be promising targets for anti-parasitic chemotherapy [39], [40]. In this study, we identified a significant increase in NO production through activation of TLR4/NF-κB signaling in epithelial cells following *C. parvum* infection. Importantly, miR-27b-regulated KSRP suppression contributes to epithelial production of NO through stabilizing iNOS mRNA, representing a new avenue to the regulation of epithelial antimicrobial defense.

Previous studies demonstrated that activation of TLR signaling can activate transcription of the iNOS gene to induce expression of iNOS [29]. We detected an increase in iNOS expression, both at the mRNA and protein levels, in cells following *C. parvum* infection *in vitro* and *in vivo* using several epithelial cell lines and a mouse model of biliary cryptosporidiosis. Our analysis also revealed an increase in iNOS mRNA stability in infected cells. Interestingly, stabilization of iNOS mRNA depends upon activation of TLR downstream signals. Inhibition of either MAPK/p38 or NF-κB signaling pathways could partially attenuate *C. parvum*-induced iNOS mRNA stabilization. Activation of the MAPK/p38 signaling pathway has been demonstrated to phosphorylate KSRP to inhibit KSRP-mediated mRNA decay [18]. Our data from this study indicate that NF-κB signaling is also involved in the regulation of iNOS mRNA stability during *C. parvum* infection. Intriguingly, activation of TLR4/NF-κB signaling by *C. parvum* promotes iNOS mRNA stability through miR-27-regulated suppression of KSRP.

The 3′UTR of human and mouse iNOS mRNA contains several AREs [19], [41], and KSRP has been shown to modulate iNOS mRNA stability by binding to the AREs within the 3′UTR and to control its half-life time in the cytoplasm [19]. Previous studies demonstrated that infection by the parasite *Trypanosoma cruzi* heightens iNOS mRNA stability in macrophages [42]. Here, we confirmed that stabilization of iNOS mRNA in epithelial cells in response to *C. parvum* infection may be ARE-dependent, since an increase in mRNA stability was shown in infected cells transfected with the luciferase ARE-construct. Furthermore, we indentified a decrease in KSRP protein level in cells following *C. parvum* infection both *in vitro* and *in vivo*. Downregulation of KSPR is TLR4/NF-κB-dependent and is through posttranscriptional mechanisms, because no significant alterations in the KSRP mRNA levels were detected in cells during *C. parvum* infection. Notably, we identified that KSRP is a target for miR-27b, and *C. parvum* infection deceases KSRP expression in host epithelial cells through upregulating miR-27b. Given the fact that the *mir-23b-27b-24-1* gene is an NF-κB-responsive gene [22], [25], we speculate that activation of TLR4/NF-κB signaling promotes iNOS mRNA stability through miR-27-regulated suppression of KSRP in cells following *C. parvum* infection. Because both TLR4 and TLR2 are recruited to the infection sites during *C. parvum* infection of H69 cells *in vitro* [4], we cannot exclude the possibility that TLR2 also may be involved in *C. parvum*-induced iNOS mRNA stabilization in biliary epithelial cells. On the other hand, miRNAs can induce RNA degradation through 3′UTR targeting, and, thus, downregulation of miRNAs should result in stabilization of targeted mRNAs [20]. Nevertheless, those miRNAs that are downregulated in H69 cells following *C. parvum* infection showed no significant complementarity with the 3′UTR of iNOS mRNA based on *in silico*-based target prediction (data not shown).

miRNA-mediated posttranscriptional mechanisms have recently been demonstrated to play an important role in regulation of epithelial innate immunity [21], [43], [44]. Cellular miRNA expression in epithelial cells can be profoundly altered during microbial infection [22]. Importantly, alterations in miRNA expression in infected cells are controlled by activation of the intracellular signaling pathway network [21]. We previously demonstrated that *C. parvum* infection activates host cell TLR4/NF-κB signaling, resulting in alterations in expression of a panel of miRNAs [22]. Functional inhibition of these NF-κB-responsive miRNAs in H69 cells increased *C. parvum* burden [22], [23], but the underlying mechanisms are still unclear. Theoretically, miRNA molecules from host cells can directly target microbial genomes and attenuate microbial replication. Indeed, several miRNAs are identified that can directly target viral genomes to restrain viral accumulation in host cells [21], [45]. Nevertheless, *C. parvum* does not express the key enzymes for the siRNA machinery [46] and therefore, regulatory RNA molecules from infected epithelial cells, including miRNAs, may have only a very limited effect on parasite biology.

In our previous studies, we identified that host epithelial cells downregulate *let-7i* to upregulate TLR4, facilitating TLR-mediated epithelial immune responses against *C. parvum* [23]. Our data from this study support the concept that TLR/NF-κB-responsive miRNAs may also target effector molecules critical to production of antimicrobial compounds in host epithelial cells, and thus, contribute to host parasitic defense. We found that miR-27b targets KSRP 3′UTR and causes translational suppression. miR-27b is upregulated in cells following *C. parvum* infection through NF-κB-regulated transactivation of the *mir-23b-27b-24-1* gene, resulting in suppression of KSRP and stabilization of iNOS RNA in infected cells. Consequently, this process promotes NO production and facilitates epithelial anti-*C. parvum* defense. Indeed, inhibition of miR-27b through anti-miR-27b and overexpression of KSRP in host cells increased *C. parvum* infection burden and hampered the eradication of *C. parvum* infection *in vitro*. Through suppression of KSRP, miR-27b also regulated *C. parvum*-induced production of other ARE-containing molecules in infected cells, such as IL-8 and COX-2. Whether other antimicrobial molecules are targets for TLR/NF-κB-responsive miRNAs merits further investigation.

Given the fact that NF-κB signaling can be activated during infection by many other pathogens, targeting of KSRP by an NF-κB-responsive miRNA (miR-27b) may have a much broader impact than just the modulation of cellular response to *C. parvum* infection. Indeed, downregulation of KSRP through upregulating miR-27b was also detected in epithelial cells following LPS stimulation. Therefore, targeting of KSRP by miR-27b and subsequent modulation of iNOS mRNA stability may be relevant to the regulation of epithelial antimicrobial defense in general.

## Materials and Methods

### Ethics statement

This study was carried out in strict accordance with the recommendations in the Guide for the Care and Use of Laboratory Animals of the National Institutes of Health under the Assurance of Compliance Number A3348-01. All animal experiments were done in accordance with procedures (protocol # 868) approved by the Institutional Animal Care and Use Committee of the Creighton University School of Medicine. All surgeries were performed under ketamine and xylazine anesthesia, and all efforts were made to minimize suffering.

### *C. parvum* and cell lines

*C. parvum* oocysts of the Iowa strain were purchased from a commercial source (Bunch Grass Farm, Deary, ID). H69 cells (a gift of Dr. D. Jefferson, Tufts University) are SV40-transformed normal human biliary epithelial cells originally derived from livers harvested for transplant. These cells continue to express biliary epithelial cell markers, including cytokeratin 19, gamma glutamyl transpeptidase, and ion transporters consistent with biliary function, and have been extensively characterized [47]. 603B cells are immortalized normal mouse biliary epithelial cells (a gift from Y. Ueno, Tohoku University School of Medicine, Sendai, Japan). HIBEpiC are non-immortalized primary human biliary epithelial cells commercially available from ScienCell Research Laboratories (Carlsbad, CA). The murine intestinal epithelial cell line (IEC4.1) was a kind gift from Pingchang Yang (McMaster University, Hamilton, Canada). SW480 and Caco-2 cells were from ATCC and were cultured according to ATCC instructions.

### Infection models and infection assay

For infection of cultured epithelial cells *in vitro*, *C. parvum* oocysts were treated with 1% sodium hypochlorite on ice for 20 min followed by extensive washing with DMEM-F12 medium. Oocysts were then excysted to release infective sporozoites (with an excystation efficiency greater than 90%), as previously reported [23]. Infection was performed in culture medium (DMEM-F12 with 100 U/ml penicillin and 100 µg/ml streptomycin) containing viable *C. parvum* sporozoites (from oocysts in a 5∶1 ratio with host cells). All experiments were performed in triplicate.

We adapted a mouse model of biliary and intestinal cryptosporidiosis via gallbladder injection of *C. parvum* originally developed by Verdon [30]. Briefly, *C. parvum* oocysts were treated with 1% sodium hypochlorite on ice for 20 min, followed by extensive washing with DMEM-F12 medium. Oocysts were then adjusted to 200,000 per 25 µl PBS and directly injected into the gallbladder of wild-type C57BL/6J or TLR4-deficient mice, as previously reported [13], [30]. *C. parvum* infection in the intrahepatic bile ducts in the wild-type and TLR4-deficient mice was observed one week and two weeks post-injection. Five animals from each group at both time points were sacrificed and liver tissues obtained for immunohistochemistry or *in situ* hybridization, as previously reported [13], [30], [48]. The C57BL/6J wild-type and TLR4-deficient (C57BL/10ScNJ; Tlr4^lps-del^) genotypes were purchased from the Jackson laboratory.

Real-time PCR and immunofluorescent microscopy were used to assay *C. parvum* infection, as previously reported [22]. Briefly, primers specific for *C. parvum* 18s ribosomal RNA (forward: 5′-TAGAGATTGGAGGTTGTTCCT-3′ and reverse: 5′-CTCCACCAACTAAGAACGGCC-3′) were used to amplify the cDNA specific to the parasite. Primers specific for human plus *C. parvum* 18s were used to determine total 18s cDNA. Data were expressed as copies of *C. parvum* 18s versus total 18s. For immunofluorescent microscopy, cells were fixed with 2% paraformaldehyde and incubated with a polyclonal antibody against *C. parvum* (a gift from Dr. Guan Zhu, Texas A&M University, College Station, TX), followed by anti-rabbit FITC-conjugated secondary antibody (Molecular Probes) and co-staining with 4′, 6-diamidino-2-phenylindole (DAPI, 5 µM) to stain cell nuclei. Labeled cells were assessed by confocal laser scanning microscopy.

### Plasmids and reagents

The expression vector for KSRP (pcDNA3-Flag-KSRP) carrying insertion of the coding regions of KSRP is a kind gift from Dr. Ching-Yi Chen (University of Alabama, Birmingham, AL). A KSRP shRNA construct was prepared in the vector pRNA-U6.1/hygro. The target sequence for KSRP was based on sequences within the KSRP coding region (GGACAGTTTCACGACAACG). The sequences are listed in Table S1. SC-514 (100 µM, Calbiochem), a potent IKK-2 inhibitor, was used to inhibit NF-κB activation [31]. SB203580 (10 µM), a specific p38 signal transduction inhibitor, was obtained from Calbiochem (San Diego, CA). Actinomycin D (10 µg/ml) was purchased from Fisher Scientific (Pittsburgh, PA). At the utilized concentrations, no cytotoxic effects of any of the chemicals were observed on H69 and 603B cells (data not shown).

### Nitrite measurements

Cells were seeded in 24-well tissue culture plates at 1.0 Χ 10^6^ cells per well and incubated in the presence or absence of *C. parvum* sporozoites (from oocysts in a 5∶1 ratio with host cells) for 24 h. For time course assays, the time points examined were 4, 8, 12, 24, and 48 h. At the end of each time point, cells were collected and nitrite was measured using the Griess reagent kit (Promega) with NaNO_2_ as the standard, as previously described [49].

### Real-time PCR

For real-time PCR analysis of mature miRNAs, total RNAs were extracted using the mirVana miRNA Isolation kit (Ambion). An amount of 0.05 µg total RNA was reverse-transcribed using the Taqman MicroRNA Reverse Transcription Kit (Applied Biosystems). Comparative real-time PCR was performed in triplicate using the Taqman Universal PCR Master Mix (Applied Biosystems) on the Applied Biosystems 7500 FAST real-time PCR System. Mature miRNA-specific primers and probes were obtained from Applied Biosystems. Normalization was performed using RNU6B primers and probes. Relative expression was calculated using the comparative CT method [22], [25].

For analysis of pri-miRNAs and mRNA, total RNA was isolated from cells with Trizol reagent (Ambion). RNAs were treated with the DNA-free Kit (Ambion) to remove any remaining DNA. Comparative real-time PCR was performed using the SYBR Green PCR Master Mix (Applied Biosystems). Specific primers for pri-miRNAs and mRNA are listed in Table S1. All reactions were run in triplicate. The Ct values were analyzed using the comparative Ct (ΔΔCt) method and the amount of target was obtained by normalizing to the endogenous reference and relative to the control (non-treated cells) [25].

### Measurement of RNA stability

H69 cells were transfected with miR-27b precursor (30 nM) or anti-miR-27b (30 nM) for 24 h, then treated with LPS (1 µg/ml) for 2 h. Transcription was stopped using actinomycin D (10 µg/ml), and RNAs were prepared at various time points following actinomycin D treatment. Real-time PCR was then performed using 500 ng of template cDNA from the resultant RNA. Each sample was run in triplicate. The relative abundance of each mRNA was calculated using the ΔΔCt method and normalized to GAPDH. The relative amount of mRNA at 0 h following actinomycin D treatment was arbitrarily set to 1. Curve fittings of the resultant data were performed using Microsoft Excel and the half-lives of selected RNAs calculated, as previously reported [37].

### Western blot

Whole cell lysates were obtained from cells with MPER mammalian protein extraction reagent (Pierce) containing several protease inhibitors (1 mM PMSF, 10 µg/ml leupeptin, and 2 µg/ml pepstatin). Cell lysates were then loaded in SDS-PAGE gel to separate proteins and transferred to nitrocellulose membrane. Abs to KSRP (Bethyl Laboratories), iNOS (Abcam), TTP (Santa Cruz Biotechnology), HuR (Santa Cruz Biotechnology), and actin (Sigma-Aldrich) were used. Densitometric levels of Western blot signals were quantified and expressed as their ratio to actin.

### *In situ* hybridization for detection of miRNA-27b

Paraffin tissue sections were deparaffinized and treated with 10 µg/ml proteinase K (Roche) at 37°C for 10 min, as reported [48]. After washing with PBS, slides were incubated with the hybridization buffer (50% formamide, 100 µg/ml salmon sperm DNA, 200 µg/ml yeast tRNA, 600 mM NaCL, 1×Denhardt's solution, 0.25% SDS, 1 mM EDTA) at 42°C for 1 h. Slides were then hybridized with 20 nM DIG-labeled miR-27b probe (Exiqon) diluted in the hybridization buffer at 42°C overnight. Slides were incubated with anti-DIG-POD Fab fragments (Roche) at 4°C overnight, and miR-27b was visualized in a staining reaction with Renaissance Tyramide Signal Amplification (TSA) Fluorescence Systems (PerkinElmer). In all experiments, a negative control, i.e., staining without miR-27b probe, was included.

### Luciferase reporter constructs and luciferase assay

Complementary DNA oligonucleotides containing the putative miR-27b target site within the 3′UTR of human KSRP (43-mer) and mouse KSRP (41-mer) were synthesized with flanking *Spe*I and *Hin*dIII restriction enzyme digestion sites (Table S1). The sense and antisense strands of the oligonucleotides were annealed. The annealed oligonucleotides were ligated into the *Spe*I-*Hin*dIII sites of the pMIR-REPORT Luciferase vector (Ambion). In these plasmids, the posttranscriptional regulation of luciferase was potentially regulated by miRNA interactions with the KSRP 3′UTR. Another pMIR-REPORT Luciferase construct containing KSRP 3′UTR with two mutants (both GTGA to ACAG) at the putative seed region for human and mouse was also generated as a control. We then transfected cells in culture with each reporter construct, as well as miR-27b antisense oligonucleotide or precursor, followed by assessment of luciferase activity 24 h after transfection. Luciferase activity was then measured and normalized to the expression of the control β-gal construct [26]. In addition, the ARE sequences (AACCUAUUUAUUAUUUAUGUAUUUAUUUA) were cloned and ligated into the pMIR-REPORT Luciferase vector for the measurement of ARE-mediated RNA stability, as previously reported [18].

## Supporting Information

Figure S1 ***C. parvum*** **infection induces stabilization of iNOS mRNA in 603B cells.** Cells were infected with *C. parvum* for 24 h. Actinomycin D (Act D) was then added and cells were collected for real-time PCR analysis. The stability of iNOS mRNAs was calculated, presented as the relative amount of mRNA to cells before Act D treatment. *, *p*<0.05 vs non-infected cells.
(TIF)

Figure S2 ***C. parvum*** **Infection decreases expression of KSRP protein without a change in mRNA level in epithelial cells.** A to C: 603B, SW480, HIBEpiC, and IEC4.1 cells were exposed to *C. parvum* for up to 24 h, followed by Western blot for KSRP protein and by real-time PCR for KSRP mRNA. D: Effects of *C. parvum* infection on the subcellular distribution of KSRP in H69 cells. Cells were exposed to *C. parvum* for 24 h and nuclear and cytoplasmic extracts were obtained and assessed by Western blot for KSRP. Representative Western blot gels from three independent experiments are shown. Actin was also blotted to ensure equal loading, and densitometric levels of KSRP signals were quantified and expressed as the ratio to actin. *, *p*<0.05 vs non-infected cells.
(TIF)

Figure S3 **miR-27b targets KSRP 3′UTR, causing translational suppression in 603B cells.** A and B: Targeting of KSRP 3′UTR results in translational suppression in 603B cells. Cells were transfected with the pMIR-REPORT luciferase construct containing the miR-27b binding site in KSRP 3′UTR and treated with the anti-miR-27b or miR-27b precursor for 24 h, followed by luciferase analysis. C and D: Manipulation of miR-27b function results in reciprocal alterations in KSRP protein expression in 603B cells. Cells were treated with various doses of miR-27b precursor or anti-miR-27b for 48 h, followed by Western blot for KSRP protein and real-time PCR for KSRP mRNA. Representative Western blot gels from three independent experiments are shown. Densitometric levels of KSRP signals were quantified and expressed as their ratio to actin. *, p<0.05 *t-*test vs. the controls.
(TIF)

Figure S4 **Anti-miR-27b inhibits downregulation of KSRP protein in SW480 cells induced by** ***C. parvum*** **.** SW480 cells were transfected with anti-miR-27b or anti-miR-control for 48 h and then exposed to *C. parvum* for 24 h, followed by Western blot for KSRP. Representative Western blot gels are shown, and densitometric levels of KSRP signals were quantified and expressed as their ratio to actin. *, p<0.05 *t*-test vs. non-infected cells.
(TIF)

Figure S5 **Functional manipulation of miR-27b affects iNOS mRNA stability in H69 cells.** Effects of miR-27b precursor or anti-miR-27b on iNOS mRNA stability in H69 cells. H69 cells were transfected with miR-27b precursor or anti-miR-27b for 48 h. The stability of mRNAs was calculated in cells following LPS stimulation for 2 h. *, p<0.05 *t-*test vs. the controls.
(TIF)

Table S1 **Listed in this table are all the primers and DNA oligonucleotides we used in this study for the real-time PCR and construct generating.** ^a^Restriction enzyme sites were indicated by lower case letters.
(TIF)
