# Supplementary figures and images for: miR-27b Targets KSRP to Coordinate TLR4-Mediated Epithelial Defense against Cryptosporidium parvum Infection
Source: PLoS Pathog. 2012 May 17;8(5):e1002702. doi: 10.1371/journal.ppat.1002702 (PMC3355088; doi:10.1371/journal.ppat.1002702)

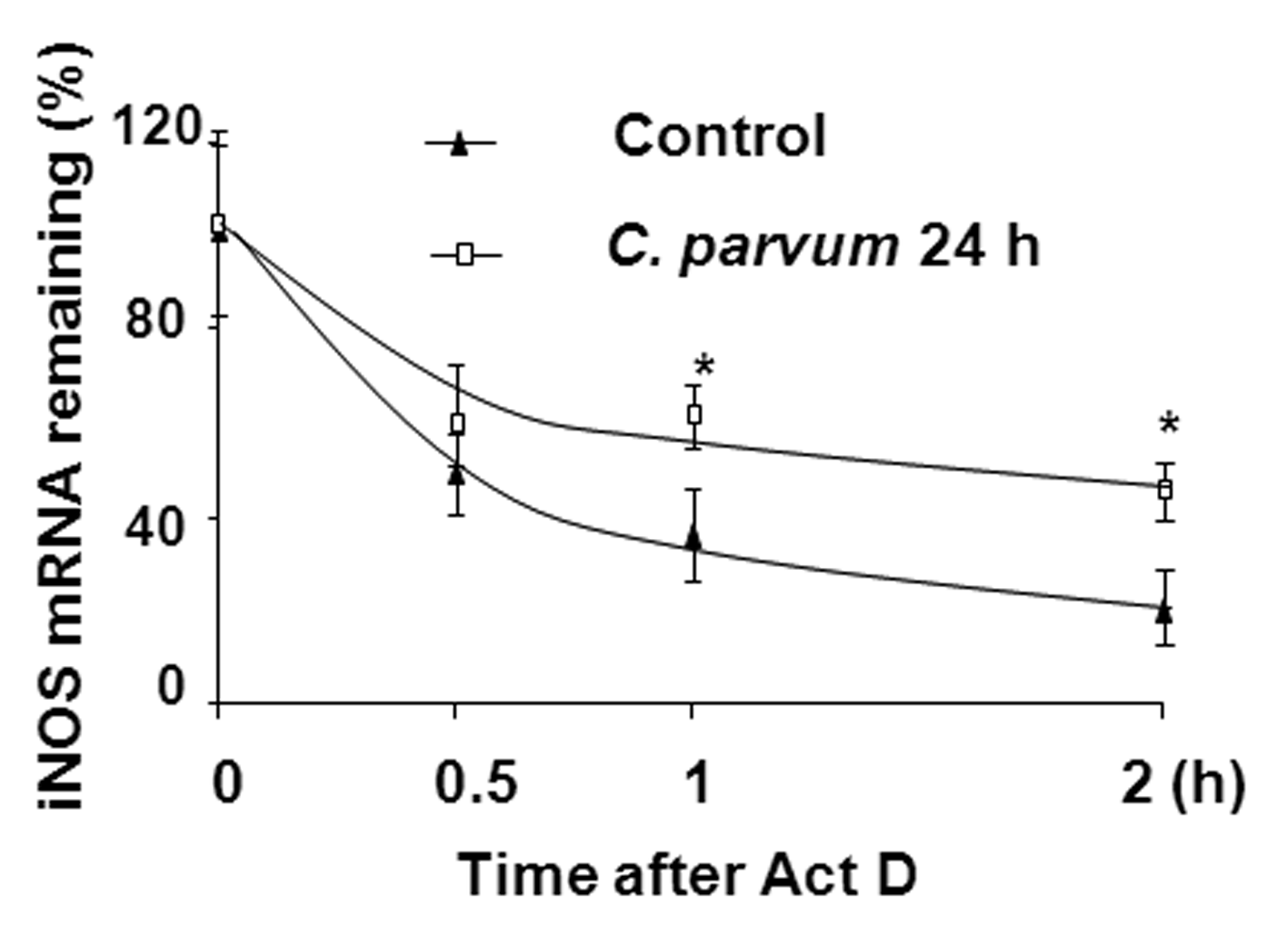

Supplement: Figure S1 — C. parvum infection induces stabilization of iNOS mRNA in 603B cells. Cells were infected with C. parvum for 24 h. Actinomycin D (Act D) was then added and cells were collected for real-time PCR analysis. The stability of iNOS mRNAs was calculated, presented as the relative amount of mRNA to cells before Act D treatment. *, p<0.05 vs non-infected cells. (TIF) [file ppat.1002702.s001.tif]

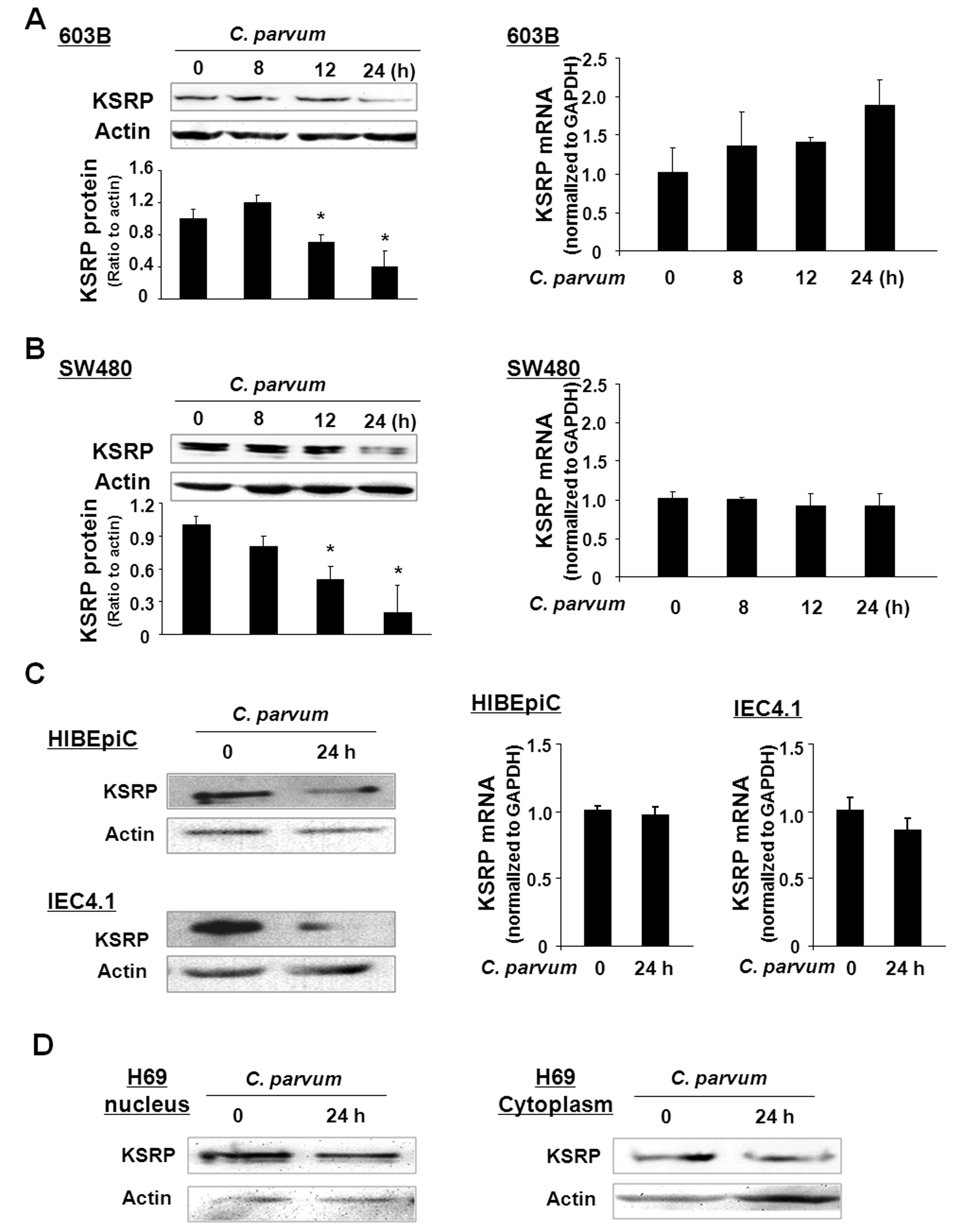

Supplement: Figure S2 — C. parvum Infection decreases expression of KSRP protein without a change in mRNA level in epithelial cells. A to C: 603B, SW480, HIBEpiC, and IEC4.1 cells were exposed to C. parvum for up to 24 h, followed by Western blot for KSRP protein and by real-time PCR for KSRP mRNA. D: Effects of C. parvum infection on the subcellular distribution of KSRP in H69 cells. Cells were exposed to C. parvum for 24 h and nuclear and cytoplasmic extracts were obtained and assessed by Western blot for KSRP. Representative Western blot gels from three independent experiments are shown. Actin was also blotted to ensure equal loading, and densitometric levels of KSRP signals were quantified and expressed as the ratio to actin. *, p<0.05 vs non-infected cells. (TIF) [file ppat.1002702.s002.tif]

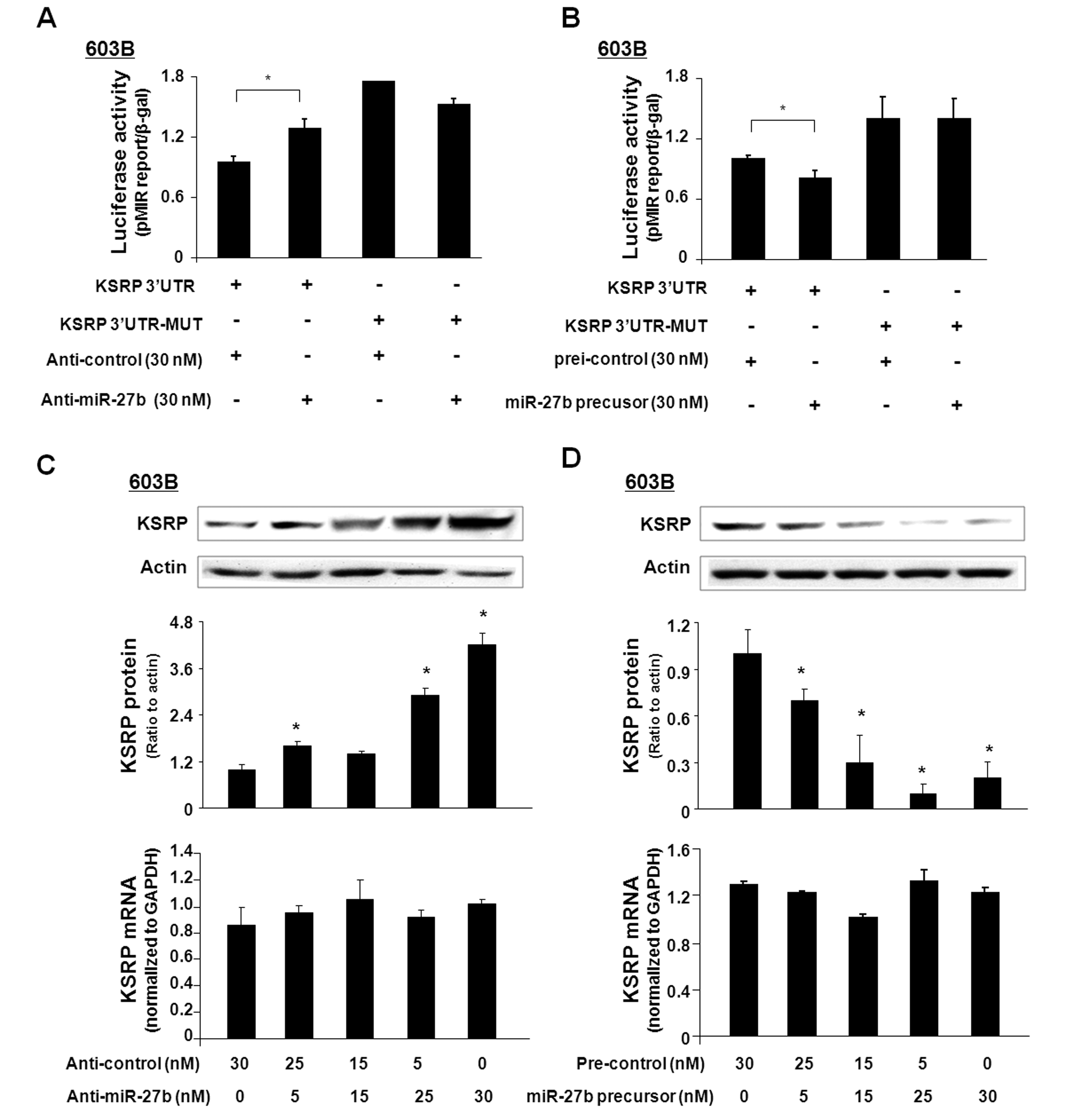

Supplement: Figure S3 — miR-27b targets KSRP 3′UTR, causing translational suppression in 603B cells. A and B: Targeting of KSRP 3′UTR results in translational suppression in 603B cells. Cells were transfected with the pMIR-REPORT luciferase construct containing the miR-27b binding site in KSRP 3′UTR and treated with the anti-miR-27b or miR-27b precursor for 24 h, followed by luciferase analysis. C and D: Manipulation of miR-27b function results in reciprocal alterations in KSRP protein expression in 603B cells. Cells were treated with various doses of miR-27b precursor or anti-miR-27b for 48 h, followed by Western blot for KSRP protein and real-time PCR for KSRP mRNA. Representative Western blot gels from three independent experiments are shown. Densitometric levels of KSRP signals were quantified and expressed as their ratio to actin. *, p<0.05 t-test vs. the controls. (TIF) [file ppat.1002702.s003.tif]

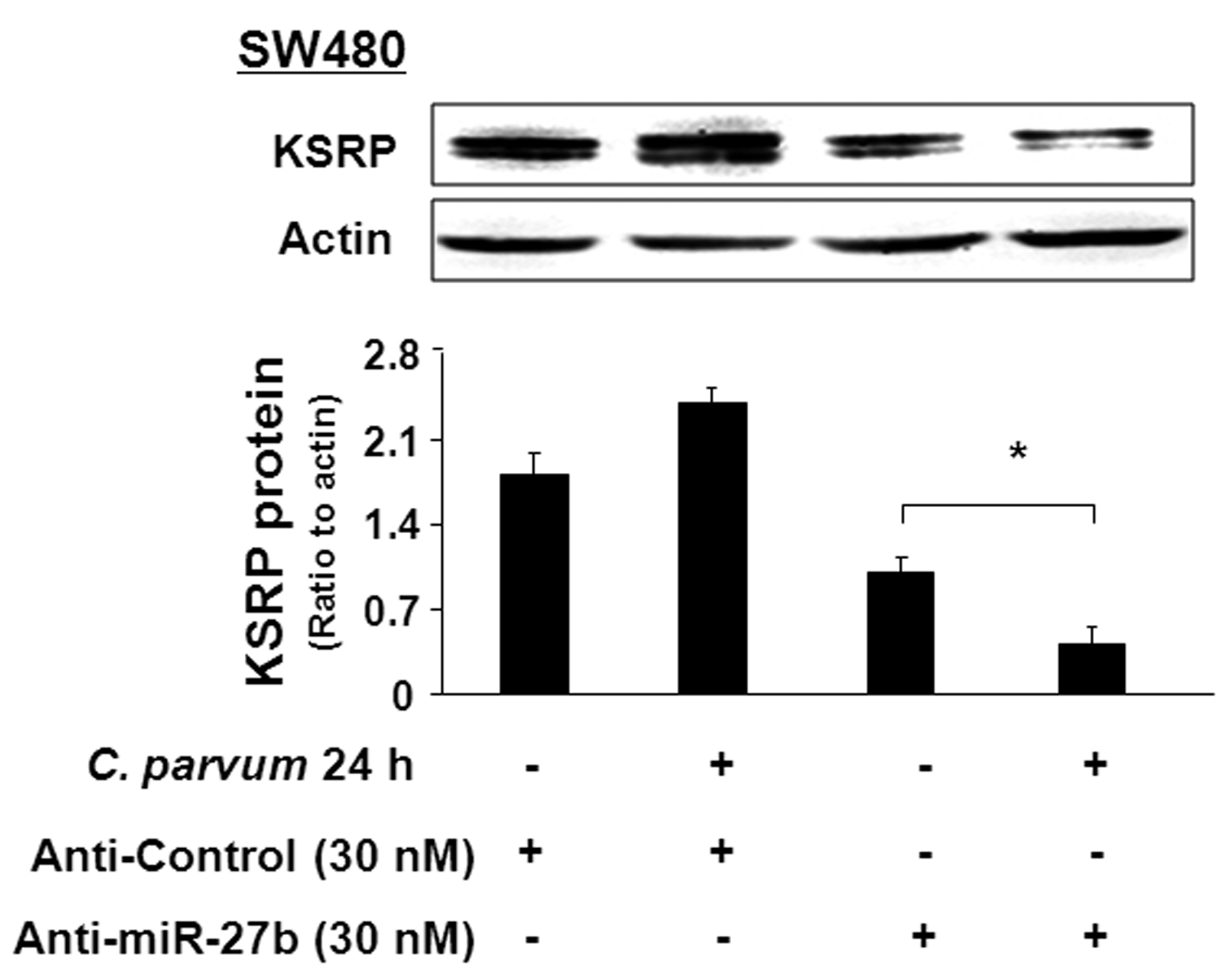

Supplement: Figure S4 — Anti-miR-27b inhibits downregulation of KSRP protein in SW480 cells induced by C. parvum . SW480 cells were transfected with anti-miR-27b or anti-miR-control for 48 h and then exposed to C. parvum for 24 h, followed by Western blot for KSRP. Representative Western blot gels are shown, and densitometric levels of KSRP signals were quantified and expressed as their ratio to actin. *, p<0.05 t-test vs. non-infected cells. (TIF) [file ppat.1002702.s004.tif]

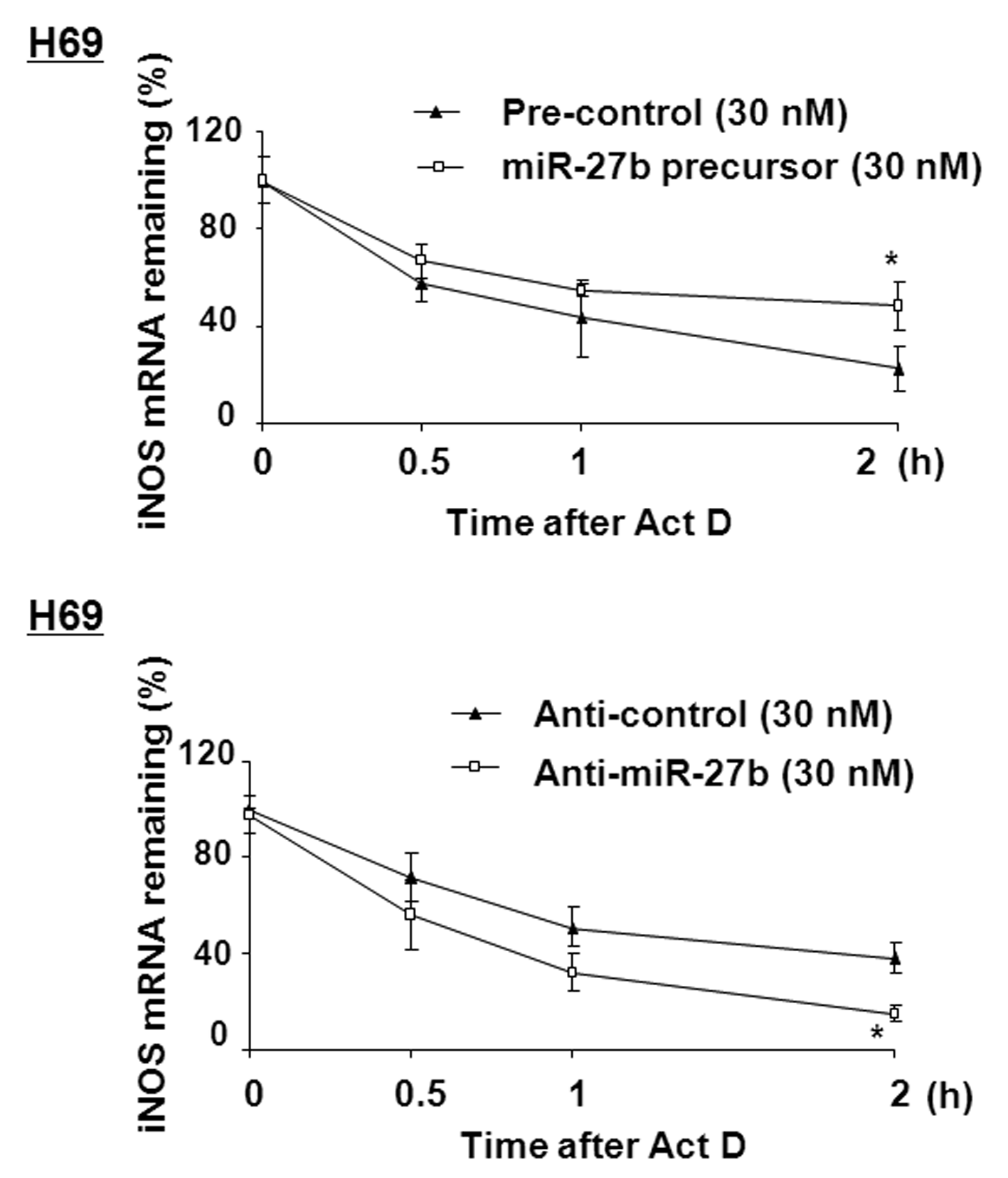

Supplement: Figure S5 — Functional manipulation of miR-27b affects iNOS mRNA stability in H69 cells. Effects of miR-27b precursor or anti-miR-27b on iNOS mRNA stability in H69 cells. H69 cells were transfected with miR-27b precursor or anti-miR-27b for 48 h. The stability of mRNAs was calculated in cells following LPS stimulation for 2 h. *, p<0.05 t-test vs. the controls. (TIF) [file ppat.1002702.s005.tif]

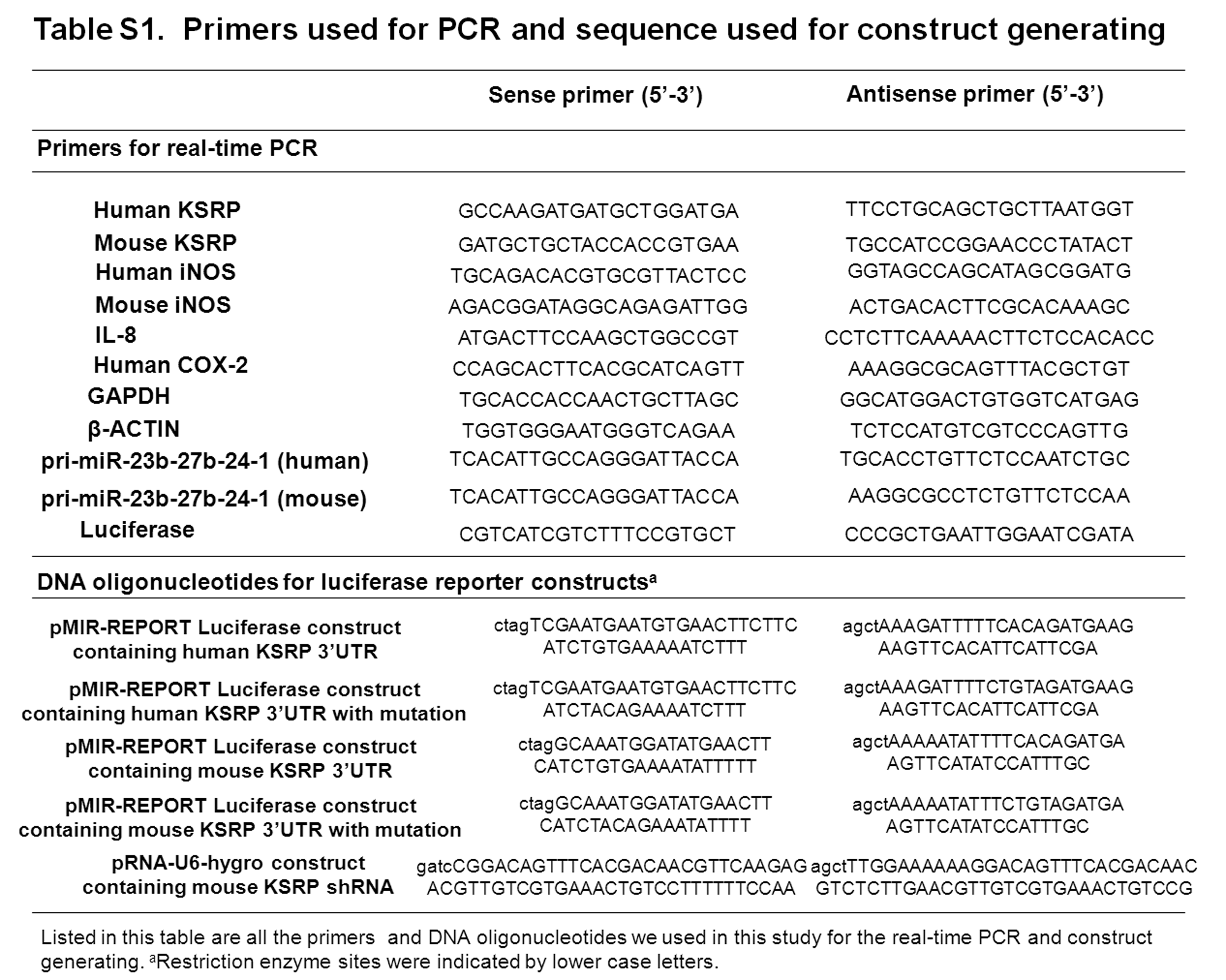

Supplement: Table S1 — Listed in this table are all the primers and DNA oligonucleotides we used in this study for the real-time PCR and construct generating. aRestriction enzyme sites were indicated by lower case letters. (TIF) [file ppat.1002702.s006.tif]
